# Aqueous Extract of *Paeonia lactiflora* and Paeoniflorin as Aggregation Reducers Targeting Chaperones in Cell Models of Spinocerebellar Ataxia 3

**DOI:** 10.1155/2013/471659

**Published:** 2013-02-25

**Authors:** Kuo-Hsuan Chang, Wan-Ling Chen, Li-Ching Lee, Chih-Hsin Lin, Pin-Jui Kung, Te-Hsien Lin, Yi-Ci Wu, Yih-Ru Wu, Yi-Chun Chen, Guey-Jen Lee-Chen, Chiung-Mei Chen

**Affiliations:** ^1^Department of Neurology, Chang Gung Memorial Hospital, Chang Gung University College of Medicine, Taipei 10507, Taiwan; ^2^Department of Life Science, National Taiwan Normal University, Taipei 11677, Taiwan

## Abstract

Spinocerebellar ataxia (SCA) types 1, 2, 3, 6, 7, and 17 as well as Huntington's disease are a group of neurodegenerative disorders caused by expanded CAG repeats encoding a long polyglutamine (polyQ) tract in the respective proteins. Evidence has shown that the accumulation of intranuclear and cytoplasmic misfolded polyQ proteins leads to apoptosis and cell death. Thus suppression of aggregate formation is expected to inhibit a wide range of downstream pathogenic events in polyQ diseases. In this study, we established a high-throughput aggregation screening system using 293 ATXN3/Q_75_-GFP cells and applied this system to test the aqueous extract of *Paeonia lactiflora* (*P. lactiflora*) and its constituents. We found that the aggregation can be significantly prohibited by *P. lactiflora* and its active compound paeoniflorin. Meanwhile, *P. lactiflora* and paeoniflorin upregulated HSF1 and HSP70 chaperones in the same cell models. Both of them further reduced the aggregation in neuronal differentiated SH-SY5Y ATXN3/Q_75_-GFP cells. Our results demonstrate how *P. lactiflora* and paeoniflorin are likely to work on polyQ-aggregation reduction and provide insight into the possible working mechanism of *P. lactiflora* in SCA3. We anticipate our paper to be a starting point for screening more potential herbs for the treatment of SCA3 and other polyQ diseases.

## 1. Introduction

Spinocerebellar ataxias (SCAs) are a large, complex group of heterogeneous autosomal dominant neurodegenerative disorders characterized by cerebellar dysfunction alone or in combination with other neurological abnormalities [1]. Among them, the expansions of CAG trinucleotide repeats encoding a polyglutamine (polyQ) stretch have been shown to cause dominantly inherited SCA1, SCA2, SCA3, SCA6, SCA7, SCA17, and dentatorubropallidoluy-sianatrophy (DRPLA) [2–8]. These polyQ-mediated genetic disorders in SCAs have shown selective progressive degeneration of the cerebellum, brainstem, and spinal tract, with prominent pathological hallmark of intranuclear and cytoplasmic accumulation of aggregated polyQ proteins inside degenerated neurons [9]. Different polyQ tract-containing proteins ultimately lead to the dysfunction and degeneration of specific neuronal subpopulations [10]. The aggregated polyQ proteins may cause dysfunction of mitochondria, chaperone, and ubiquitin proteasome system, leading to apoptosis and cell death [11–13]. As misfolding of the polyQ protein is likely the initial event in the pathogenic cascade, suppression of protein misfolding is expected to inhibit a wide range of downstream detrimental events, and to rescue neuronal dysfunction.

Increasing evidence suggests that some herbs may potentially attenuate the deterioration of neurodegenerative diseases. *Paeonia lactiflora *(*P. lactiflora*), belonging to the Paeoniaceae family, is a perennial herb frequently used as an important ingredient in many traditional prescriptions. It has been commonly used for nourishing blood, alleviating pain, reducing irritability, as well as treating liver disease and cancer [14]. Paeoniflorin, one of the main compounds extracted from *P. lactiflora*, has been reported to ameliorate neurodegenerative process in Parkinson's disease (PD) and Alzheimer's disease (AD) models [15–17]. However, the effect of *P. lactiflora *herb extract and paeoniflorin in treating SCA remains unraveled.

In the present study, we firstly built up an aggregation screening cell model by overexpressing CAG-expanded ATXN3, the causative mutation in SCA3 [2], in 293 cells, and then examined the anti-aggregation effect of *P. lactiflora* aqueous extract and paeoniflorin. We further demonstrated that the anti-aggregation activity of *P. lactiflora* extract and paeoniflorin was contributed by the enhancement of heat shock transcription factor 1 (HSF1)-heat shock protein (HSP) 70 chaperone system. These findings provide evidence that *P. lactiflora* and paeoniflorin may be a novel alternative therapeutic agent for the treatment of SCAs.

## 2. Materials and Methods

### 2.1. *P. lactiflora* Extract Preparation and HPLC Analysis

Aqueous extract from *P. lactiflora* was provided by Sun-Ten Pharmaceutical Company (Taipei, Taiwan). Briefly, 100 g of dried *P. lactiflora* was boiled with 1500 mL of water at 100°C for 30 min and was sieved using a 100-mesh sieve. The extract was concentrated to 100 mL and filtered through a 200-mesh sieve. The extract was then dried by speed vacuum concentration and then stored at −20°C until used. 

High pressure liquid chromatography (HPLC) was performed using a LaChrom Elite HPLC system (Hitachi), consisting of a photo diode array detector. The chromatographic separation of *P. lactiflora* extract (50 *μ*L, 1 mg/mL) was carried out on a Hypersil ODS (C18) column (250 × 4.6 mm, 5 *μ*m), eluted with the mixture of 0.1% formic acid in water (A) or acetonitrile (B). The linear gradient elution program for A : B (v/v) was set as follows: 95 : 5 (0–10 min), 95 : 5–70 : 30 (10–40 min), 70 : 30–15 : 85 (40–55 min), 15 : 85–95 : 5 (55–60 min), 95 : 5 (60–75 min) with a flow rate of 1 mL/min. Absorbance was monitored at 230, 250, 270 nm and the scan range for photo diode array was 190*∼*400 nm. Paeoniflorin, gallic acid, and albiflorin (2*∼*10 *µ*L, 20 mM) were used as reference compounds for *P. lactiflora* [18, 19].

### 2.2. Cell Culture and Cell Proliferation Assay

Human embryonic kidney HEK-293 cells (ATCC No. CRL-1573) were cultivated in Dulbecco's modified Eagle's medium (DMEM) containing 10% fetal bovine serum (FBS). Human neuroblastoma SH-SY5Y cells (ATCC No. CRL-2266) were maintained in DMEM F12 supplemented with 10% FBS. Cells were cultivated at 37°C incubator containing 5% CO_2_ and cell proliferation was measured based upon the reduction of the tetrazolium salt, 3,[4,5-dimethylthiazol-2-yL]-2,5-diphenyl-tetrazolium bromide (MTT). Cells were plated into 48-well (5 × 10^4^/well) dishes, grown for 20 hr and treated with different concentrations of the *P. lactiflora* extract (5*∼*30 mg/mL) or pure compound (100 nM*∼*1 mM). After one day, 20 *μ*L MTT (5 mg/mL in PBS, Sigma) was added to cells and incubated for 2 hr. The absorbance of the purple formazan dye was measured at 570 nm by a Bio-Tek *μ*Quant Universal Microplate Spectrophotometer.

### 2.3. Flp-In-293 Triple Fluorescent Reporter Cells and Fluorescent Assay

A triple fluorescent reporter plasmid with mCherry, ZsYellow1, and AmCyan1 was first constructed in pAmCyan1-N1. A proximal promoter (−360*∼*+2, with the translation initiation site as +1) from HSF1 gene was used, as 331 bp upstream of the murine HSF1 translation start site is required for maximal basal expression [20]. Promoter fragments from HSF1 (enhancing chaperone expression), heat shock cognate protein (HSPA8, −1140*∼*+38, driving constitutively expressed HSP70) [21] and heat-inducible HSP70 chaperone (HSPA1A, −273*∼*+215, driving heat-inducible HSP70) [22] are placed upstream of the three fluorescent reporters. The fragment containing the HSF1, HSPA8, and HSPA1A driven reporters was excised with *Ase*I and *Not*I restriction enzymes and used to replace an *Ase*I-*Not*I fragment in pcDNA5/FRT/TO plasmid (Invitrogen). The resulting triple fluorescent reporter plasmid was used to generate Flp-In triple fluorescent reporter cells and maintained according to the supplier's instructions (Invitrogen). Geranylgeranylacetone (GGA, Sigma) or paeoniflorin (100 nM~100 *µ*M) was added to the medium for 24 hr. The three fluorescence colors were analyzed simultaneously using high-content analysis (HCA) system (ImageXpressMICRO, Molecular Devices), with excitation/emission wavelengths at 453/486 (mCherry), 531/540 (ZsYellow1) and 587/610 nm (AmCyan1).

### 2.4. ATXN3 and HSF1 cDNA Constructs

Polyadenylated RNA (200 ng) isolated from neuroblastoma SK-N-SH cells was reverse transcribed using the SuperScript III reverse transcriptase (Invitrogen). The sense and antisense primers used for ATXN3/Q_14_ cDNA (+826*∼*+1152, NM_004993) amplification were 5′-ATTCAGCTAAGTATGCAAGGTAGTTCCA (codon for Met257 underlined) and 5′-CATGCCATGGCATGTTTTTTTCCTTCTGTT (*Nco*I site underlined). The amplified 3′ polyQ-containing cDNA fragment (translated into amino acids 257*∼*361) was cloned into pGEM-T Easy (Promega) and sequenced. The ATXN3/Q_14_ cDNA was excised with *Eco*RI (in pGEM-T Easy vector) and *Nco*I and subcloned into pEGFP-N1 (Clontech). Then DNA fragment containing in-frame ATXN3/Q_14_-EGFP was excised with *Hin*dIII-*Not*I and subcloned into the pcDNA5/FRT/TO. The ATXN3/Q_75_ cDNA was made by replacing an 88 bp ATXN3/Q_14_ *Bsm*BI-*Bsm*FI fragment with a 271 bp ATXN3/Q_75_ fragment from the cDNA clone of a SCA3 patient. The HSF1 cDNA (BC014638) in pOTB7 was obtained from Bioresource Collection and Research Center (BCRC), Food Industry Research and Development Institute, Taiwan. The cDNA was excised with *EcoR*I and *XhoI* and subcloned into pcDNA3 (Invitrogen).

### 2.5. Isogenic 293 and SH-SY5Y Cell Lines

Human 293-derived Flp-In-293 cells (Invitrogen) were cultivated in DMEM containing 10% FBS as described. The cloned pcDNA5/FRT/TO-ATXN3/Q_14_ and Q_75_ plasmids were used to generate the isogenic ATXN3/Q_14*∼*75_ cell lines by targeting insertion into Flp-In-293 cells, according to the supplier's instructions. The repeats in these ATXN3 cell lines were examined by PCR and sequencing. These cell lines were grown in medium containing 5 *µ*g/mL blasticidin and 100 *µ*g/mL hygromycin (InvivoGen). Human SH-SY5Y-derived Flp-In host cell line was constructed as described [23]. The SH-SY5Y host cells were used to generate isogenic ATXN3/Q_14*∼*75_ lines and maintained as described above. 

### 2.6. ATXN3/Q_75_ Aggregation Assay

293ATXN3/Q_75_-GFP cells were plated into 96-well (2 × 10^4^/well) dishes, grown for 24 hr and treated with different concentrations of the *P. lactiflora* extract (2*∼*200 *μ*g/mL) or suberoylanilide hydroxamic acid (SAHA, Cayman Chemical), paeoniflorin (Sigma), gallic acid, and albiflorin (Chromadex) (100 nM*∼*5 *µ*M) for 8 hr. Then doxycycline (10 *μ*g/mL, BD) was added to the medium to induce ATXN3/Q_75_-GFP expression for 6 days. Oxaliplatin (5 *μ*M, Sigma) was also added for aggregate accumulation through inhibition of cell division [24]. Then cells were stained with Hochest 33342 (0.1 *μ*g/mL, Sigma) and aggregation percentage was assessed by HCA system, with excitation/emission wavelengths at 482/536 (GFP).

SH-SY5Y ATXN3/Q_75_-GFP cells were seeded in 6-well (2 × 10^5^/well) plate, with all *trans* retinoic acid (10 *µ*M, Sigma) added at seeding time. At day 2, cells were treated with paeoniflorin (100** **nM) or the *P. lactiflora* extract (10 *µ*g/mL) for 8** **hr, and then doxycycline (5 *μ*g/mL) was added to induce ATXN3/Q_75_-GFP expression. The cells were kept in the medium containing 10 *μ*M trans retinoic acid, doxycycline and paeoniflorin/*P. lactiflora* extract for 7 days. After that, cells were stained with Hochest 33342 (0.1 *μ*g/mL) and aggregation percentage was assessed as described. 

### 2.7. Real-Time PCR

Total RNA from 293 ATXN3 lines was extracted using Trizol reagent (Invitrogen). The RNA was DNase (Stratagene) treated, quantified, and reverse-transcribed to cDNA as described. Real-time quantitative PCR experiments were performed in the ABI PRISM 7000 Sequence Detection System (Applied Biosystems). Amplification was performed on 100 ng cDNA with gene-specific TaqMan fluorogenic probes Hs0024525_ml for ATXN3 and 4326321E for HPRT1 (endogenous control) (Applied Biosystems). Fold change was calculated using the formula 2^ΔC*t*^, ΔC_*t*_ = C_*t*_(control) − C_*t*_(target), in which C_*t*_ indicates cycle threshold.

### 2.8. Western Blot Analysis

Total proteins were prepared using lysis buffer containing 50 mM Tris-HCl, 150 mM NaCl, 1 mM EDTA, 1 mM EGTA, 0.1% SDS and 0.5% sodium deoxycholate, 1% Triton X-100, protease inhibitor cocktail (Calbiochem). Proteins (25 *μ*g) were separated on 10% SDS-polyacrylamide gel electrophoresis and blotted on to nitrocellulose membranes by reverse electrophoresis. After blocking, the membrane was probed with HSF1 (1 : 1000 dilution, Abnova), HSPA8 (1 : 500 dilution, Santa Cruz), HSPA1A (1 : 500 dilution, Santa Cruz), H3F3B (1 : 3000 dilution, GeneTex), GFP (1 : 500 dilution, Santa Cruz), *β*-actin (1 : 5000 dilution, Millipore) or GAPDH (1 : 1000 dilution, MDBio) at 4°C overnight. Then the immune complexes were detected by horseradish peroxidase-conjugated goat anti-mouse or goat anti-rabbit IgG antibody (1 : 5000 dilution, GeneTex) and chemiluminescent substrate (Millipore).

### 2.9. ATXN3/Q_75_ and HSF1 cDNA Co-Transfection

Human embryonic kidney HEK-293T cells (ATCC No. CRL-11268) were cultivated in DMEM containing 10% FBS as described. For transient overexpression, cells were plated into 12-well (1 × 10^5^/well) dishes, grown for 20 hr, and transfected using T-Pro reagent (JF Biotechnology, Taiwan) with pcDNA5/FRT/TO-ATXN3/Q_75_ and pcDNA3-HSF1 or pcDNA3 vector plasmids (1.5 *μ*g each). The cells were grown for 48 hr for ATXN3/Q_75_ aggregation assay as described.

### 2.10. Statistical Analysis

For each set of values, data were expressed as the means ± standard deviation (SD). Three independent experiments were performed and non-categorical variables were compared using the Student's *t*-test. All *P* values were two-tailed, with values of *P* < 0.05 considered significant.

## 3. Results

### 3.1. Construction of 293 Cells Expressing ATXN3/Q_75_ Aggregates

**Figure 1:**
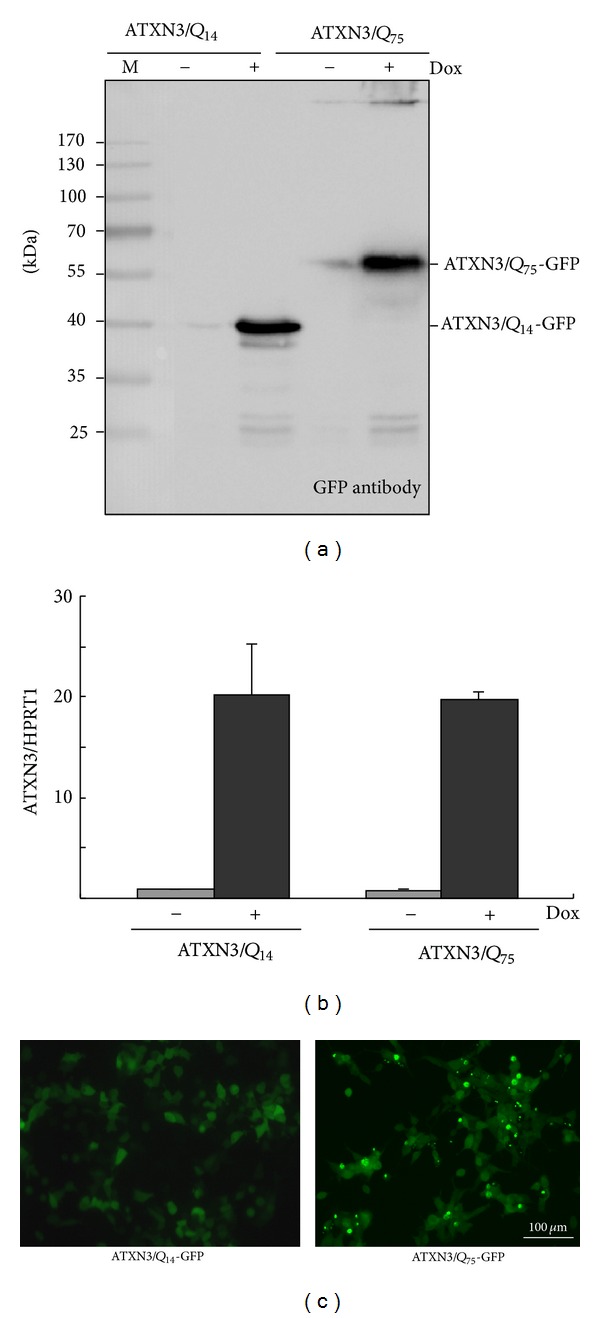
Flp-In 293 cells with ATXN3/Q_14*∼*75_-GFP expression in an inducible fashion. (a) Western blot analysis of ATXN3/Q_14*∼*75_-GFP protein expression using GFP antibody after two days of induction (+Dox). (b) Real-time PCR quantification of ATXN3/Q_14*∼*75_-GFP RNA expression relatively to HPRT after two days of induction (+Dox). (c) Fluorescence microscopy images of ATXN3/Q_14*∼*75_-GFP expression after six days of induction. The scale bar = 100 *µ*m.

For therapies toward the polyQ diseases, we aimed to screen herbs/compounds potentially inhibiting polyQ aggregation. As removal of the N-terminus of polyQ-expanded ATXN3 is required for aggregation *in vitro* and *in vivo *[25], we cloned GFP-tagged ATXN3 C-terminal Q_14*∼*75_-containing fragment to establish Flp-In 293 cells with ATXN3/Q_14*∼*75_-GFP expression in an inducible fashion. As shown in Figure 1(a), the GFP antibody detected 40 kDa ATXN3/Q_14_-GFP and 57 kDa ATXN3/Q_75_-GFP proteins in doxycycline (Dox) induced ATXN3 cells. ATXN3-RNA levels were then examined by real-time PCR using ATXN3-specific probe and primers. As shown in Figure 1(b), in the presence of Dox, the two ATXN3 lines expressed *∼*20 times more ATXN3 RNA than in the absence of Dox. While the expressed ATXN3/Q_14_ was mainly diffused, the expressed ATXN3/Q_75_-GFP formed aggregates (Figure 1(c)).

### 3.2. Aqueous Extract of *P. lactiflori* and Constituents

**Figure 2:**
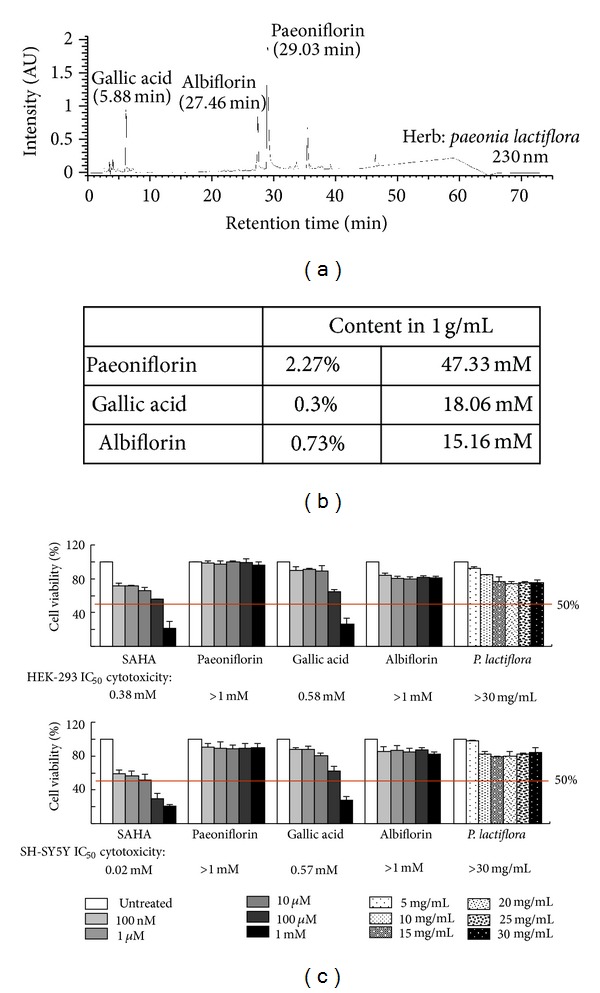
Chemical profile and cytotoxicity of the aqueous extract of *P. lactiflora. *(a) Chromatographic patterns from HPLC analysis (230 nm) showed peaks compatible with paeoniflorin, albiflorin and garlic acid. (b) The relative amount of above molecules in the extract. (c) Cytotoxicity of the aqueous extract of *P. lactiflora*, paeoniflorin, garlic acid, albiflorin, and SAHA against HEK-293 and SH-SY5Y cells using MTT viability assay. The IC_50_ of each herb/compound was shown under the columns. To normalize, the relative viability in untreated cells is set as 100%. The red line represents 50% viability.

To examine the potential active compounds in *P. lactiflori*, the chemical profile of aqueous extract was analyzed and quantified by full-spectrum analytic HPLC. Chromatographic patterns showed peaks in 230 nm corresponding to the retention time compatible with paeoniflorin, garlic acid and albiflorin (Figure 2(a)). The amounts of paeoniflorin, garlic acid, and albiflorin in aqueous extract of* P. lactiflori *were 2.27%, 0.30%, and 0.73%, respectively, corresponding to 47.33 mM, 18.06 mM, and 15.16 mM, respectively, in 1 g/mL aqueous extract (Figure 2(b)).

MTT assays were performed with human embryonic kidney 293 and human neuroblastoma SH-SY5Y cells after treatment with extract of *P. lactiflora* and the its three constituents, respectively, for 24 hr. The histone deacetylase inhibitor suberoylanilide suberoylanilide hydroxamic acid (SAHA) known to reduce SDS-insoluble polyQ aggregate [26] was included for comparison. The IC_50_ of the herb and compounds were calculated using the interpolation method. Both *P. lactiflora* extract and its constituents paeoniflorin and albiflorin had an IC_50_ higher than the highest concentration tested ( >30 mg/mL for* P. lactiflora* and >1 mM for paeoniflorin and albiflorin), suggesting their very low cytotoxicity (Figure 2(c)).

### 3.3. *P. lactiflori* Extract and Paeoniflorin Reduce ATXN3/Q_75_ Aggregation on 293 Cell Model

**Figure 3:**
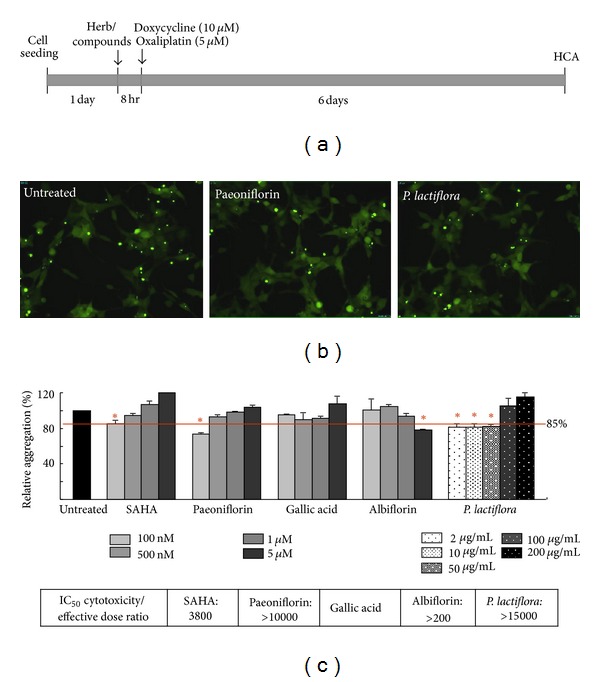
High-content compound screen using Flp-In 293 cells with inducible ATXN3/Q_75_-GFP expression. (a) Experiment flow chart. ATXN3/Q_75_-GFP 293 cells were plated into 96-well dishes, grown for 24 hr and treated with different concentrations of the herb or compound for 8 hr. Then doxycycline and oxaliplatin was added to the medium to for 6 days and aggregation percentage was assessed by HCA system. (b) Representative fluorescence microscopy images of ATXN3/Q_75_-GFP cells untreated or treated with *P. lactiflora* (10 *µ*g/mL) or paeoniflorin (100 nM) for 6 days. (c) Aggregation analysis of ATXN3/Q_75_-GFP cells untreated or treated with aqueous extract of *P. lactiflora* (2*∼*200 *µ*g/mL), paeoniflorin, garlic acid, albiflorin and SAHA (100 nM*∼*5 *µ*M). To normalize, the relative aggregation level in untreated cells is set as 100%. The red line represents 85% aggregation with SAHA treatment (100 nM).

To screen if herb/compounds potentially inhibit aggregation, we used ATXN3/Q_75_-GFP cells to examine aqueous extract of *P. lactiflora* and its constituents for their potentials to reduce the ATXN3/Q_75_ aggregation. The experiment flow chart is shown in Figure 3(a) and representative fluorescence microscopy images of aggregation after treatment with paeoniflorin and the aqueous extract of* P. lactiflora* are shown in Figure 3(b). As a positive control, HDAC inhibitor SAHA reduced the ATXN3/Q_75_ aggregation to 85% (at 100 nM) as compared to untreated cells (Figure 3(c)). While garlic acid did not display good aggregation-inhibitory potential (90*∼*95% at 100 nM*∼*1**** *μ*M), *P. lactiflora *(81*∼*82% at 2*∼*50**** *μ*g/mL), paeoniflorin (73% at 100 nM) and albiflorin (78% at 5 *μ*M) had greater aggregation reduction potential than SAHA (Figure 3(c)). The IC_50_ cytotoxicity/effective (reduced the ATXN3/Q_75_ aggregation to 85% or lower) dose ratio of SAHA, paeoniflorin, albiflorin, and extract of *P. lactiflora* were 3800, >10000, >200, and >15000, respectively. Considering 2**** *µ*g/ml of *P. lactiflora *extract contained 95 nM paeoniflorin and 30 nM albiflorin and tested greatest aggregation reduction potential of 100 nM for paeoniflorin and 5 *µ*M for albiflorin, paeoniflorin was regarded as a major active component for the aggregation inhibition in *P. lactiflora*.

### 3.4. Paeoniflorin Enhanced HSF1 and HSP70 Chaperone Expression on 293 Cells

**Figure 4:**
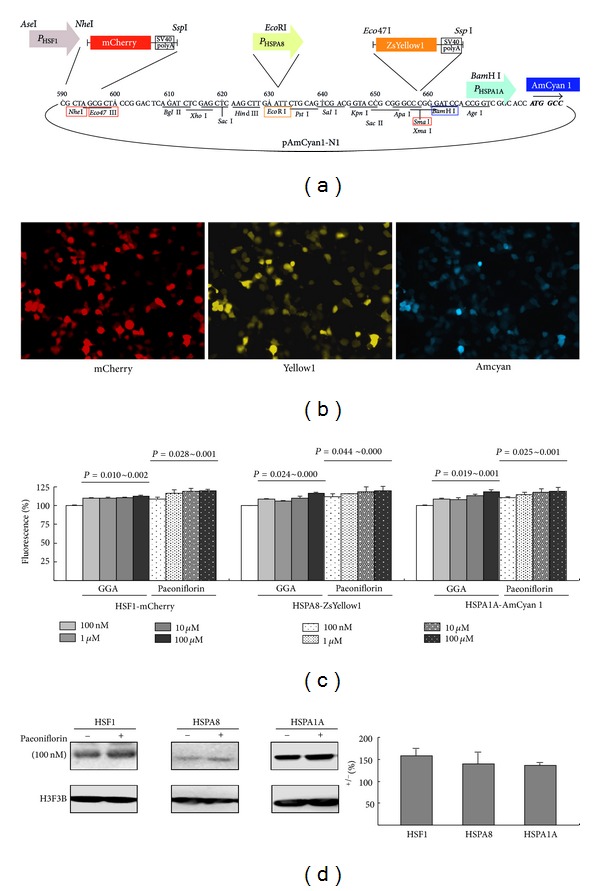
Enhancement of chaperone expression by paeoniflorin in 293 cells. (a) Triple fluorescent reporter plasmid with HSF1, HSPA8, and HSPA1A promoter fragments upstream of mCherry, ZsYellow1, and AmCyan1 fluorescent reporters, respectively. (b) Microscopic images of the triple fluorescent reporter cells. (c) Effect of GGA and paeoniflorin (100 nM*∼*100 *µ*M) on HSF1, HSPA8, and HSPA1A reporters. To normalize, the fluorescence level in untreated cells is set as 100%. Three independent experiments were performed with *P* < 0.05 considered significant. (d) Representative western blot image of paeoniflorin-(100 nM) treated 293 cells for two days using HSF1, HSPA8, HSPA1A, and H3F3B antibodies. Levels of HSF1, HSPA8, HSPA1A, were normalized with a loading control (H3F3B). Data are expressed as the mean ± SEM values from three independent experiments.

To screen the potential of herb/compounds to enhance HSF1 and HSP70 chaperone expression, we established a triple fluorescent reporter 293 cell model with mCherry, ZsYellow1 and AmCyan1 reporters downstream of HSF1, HSPA8 and HSPA1A promoters (Figure 4(a)). The cloned promoters effectively drove the expression of red, yellow, and blue fluorescent reporters (Figure 4(b)). As shown in Figure 4(c), treatment of GGA (100 nM~100 *µ*M), a potent HSP inducer, for one day significantly increased HSF1 (110%~112%, *P* = 0.010~0.002), HSPA8 (106%*∼*116%, *P* = 0.024~0.000) and HSPA1A (108%*∼*118%, *P* = 0.019~0.001) promoter activity. This was also true for paeoniflorin (100 nM*∼*100 *µ*M) treatment, with 108%*∼*120% HSF1 (*P* = 0.028~0.001), 112%*∼*120% HSPA8 (*P* = 0.044*∼*0.000) and 110%*∼*119% HSPA1A (*P* = 0.025*∼*0.001) promoter activities compared to no treament. The enhancement of paeoniflorin (100 nM) on HSF1 (158%, *P* = 0.027), HSPA8 (140%, *P* = 0.011) and HSPA1A (137%, *P* = 0.007) expression was confirmed by the Western blot in HEK-293 cells after two days treatment (Figure 4(d)).

### 3.5. *P. lactiflori* Extract and Paeoniflorin Enhanced HSF1 and HSP70 Chaperone Expression on 293 ATXN3/Q_75_ Cell Model

**Figure 5:**
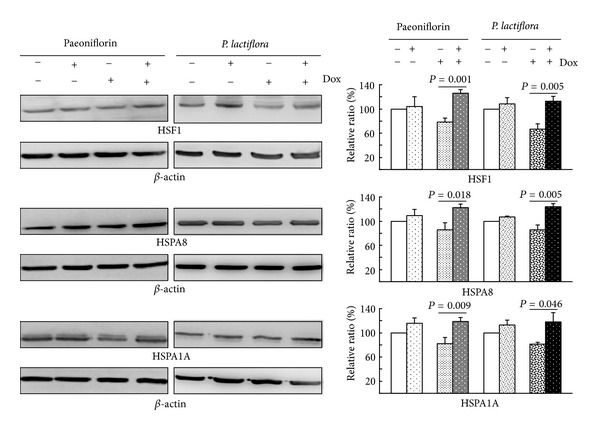
Enhancement of HSF1 and chaperone expression by paeoniflorin and the aqueous extract of *P. lactiflora* in ATXN3/Q_75_-GFP 293 cells. Cells were pre-treated with paeoniflorin (100 nM) or herb (10 *μ*g/mL) for 8 hours and ATXN3/Q_75_-GFP expression induced for 6 days. Relative HSF1, HSPA8, and HSPA1A expressions were analyzed by western blot analysis using *β*-actin as a loading control.

To examine if paeoniflorin and *P. lactiflora *extract also up-regulated HSF1 and HSP70 chaperone expression in ATXN3/Q_75_ 293 cells, we compared the expression levels of HSF1, HSPA8 and HSPA1A between with and without paeoniflorin/*P. lactiflora* and/or Dox treatment. As shown in Figure 5, induced expression of ATXN3/Q_75_ for 6 days attenuated the expression of HSF1 (78%*∼*67%), HSPA8 (86%) and HSPA1A (82%). This reduction can be rescued by the addition of paeoniflorin (100 nM) or* P. lactiflora* (10 *µ*g/mL), with significantly increased HSF1 (113%*∼*126%, *P* = 0.005*∼*0.001), HSPA8 (123%*∼*124%, *P* = 0.018*∼*0.005) and HSPA1A (118%*∼*119%, *P* = 0.046*∼*0.009) expression. These findings suggested that *P. lactiflora* and paeoniflorin up-regulated HSF1 and HSP70 chaperon expression to reduce ATXN3/Q_75_ aggregation in this cell model.

### 3.6. HSF1 Overexpression to Reduce ATXN3/Q_75_ Aggregation

**Figure 6:**
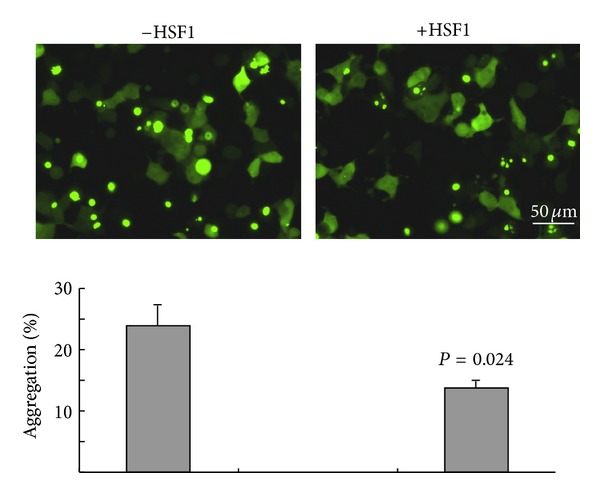
HSF1 overexpression in ATXN3/Q_75_-GFP transient cell models. HEK-293T cells were co-transfected with plasmids encoding ATXN3/Q_75_-GFP and plasmid with (+HSF1) or without (−HSF1) HSF1 cDNA. After 2 days, aggregation percentage was assessed by HCA system. (The scale bar = 50 *μ*m). The percentage of aggregate formation counted among three random fields.

To determine whether HSF1 could suppress aggregation of mutant ATXN3, we transiently co-expressed HSF1 with ATXN3/Q_75_ in HEK-293T cells. As shown in Figure 6, with HSF1 co-transfection, visible aggregates significantly decreased in ATXN3/Q_75_ cells (13.8% versus 24.0%, *P* = 0.024).

### 3.7. *P. lactiflori* Extract and Paeoniflorin Reduced ATXN3/Q_75_ Aggregation on SH-SY5Y Cell Model

**Figure 7:**
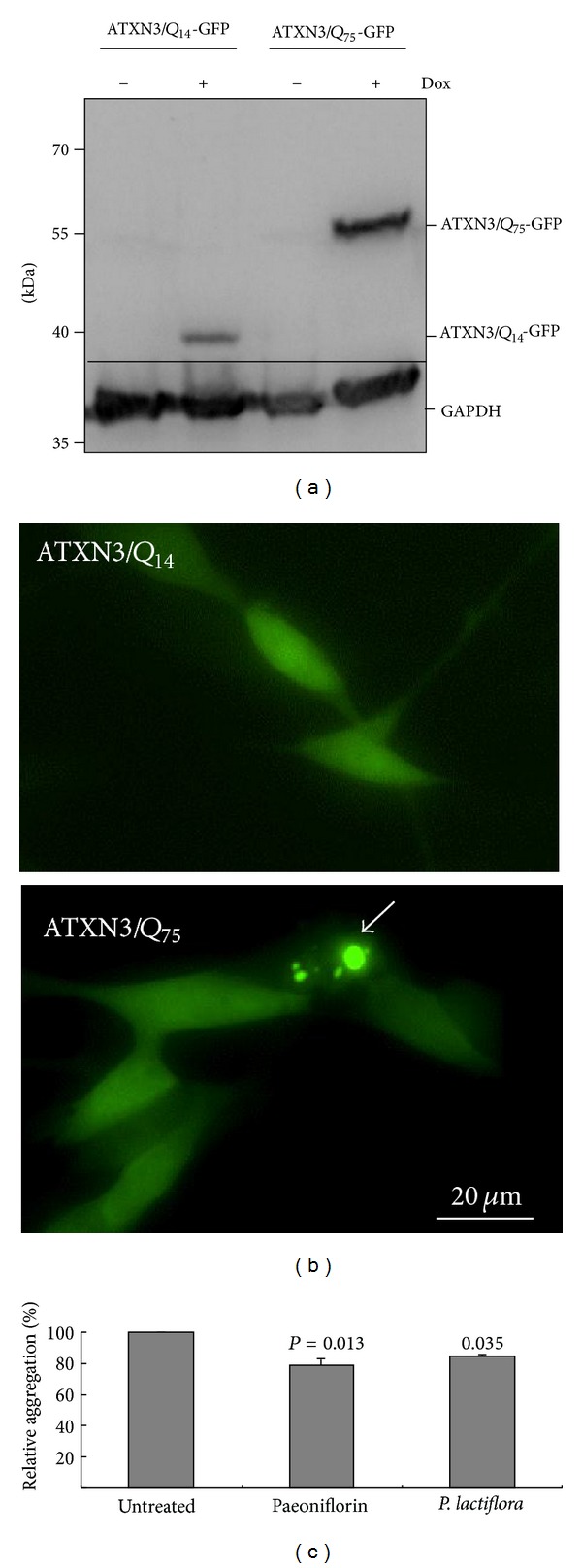
Reduction of aggregation by paeoniflorin and the aqueous extract of *P. lactiflora* in ATXN3/Q_75_-GFP SH-SY5Y cells. (a) Western blot analysis using GFP and GAPDH antibodies after two days induction (+Dox). (b) Microscopic images of differentiated SH-SY5Y cells expressing ATXN3/Q_14/75_-GFP for 6 days (the scale bar = 20 *μ*m). (c) Relative aggregation after treatment with paeoniflorin (100 nM) or the aqueous extract of *P. lactiflora* (10 *μ*g/mL) for 6 days. To normalize, the relative aggregation level in untreated cells is set as 100%.

To test the aggregation reduction potential of *P. lactiflori* extract and paeoniflorin in neuronal cells, we constructed Flp-In SH-SY5Y cells with N-terminal truncated ATXN3/Q_14*∼*75_-GFP expression in an inducible fashion. GFP-tagged 40*∼*57 kDa ATXN3/Q_14*∼*75_ protein in Dox-induced SH-SY5Y cells can be seen in Western blot (Figure 7(a)). Then we differentiated ATXN3/Q_14*∼*75_ SH-SY5Y cells using retinoic acid and found that the induced ATXN3/Q_75_ formed aggregates in *∼*1% neuronal cells (Figure 7(b)). The treatment of paeoniflorin or *P. lactiflora* leaded to 21%*∼*16% of aggregation reduction (*P* = 0.013*∼*0.035) in ATXN3/Q_75_ expressed neuronal cells (Figure 7(c)). These results confirmed the aggregation-inhibitory effect of paeoniflorin and *P. lactiflora* in differentiated neurons.

## 4. Discussion

Although Chinese herbs have been reported to reduce pneumonia risk in elderly patients with dementia [27] and regarded as a potential treatment of Huntington's disease (HD) [28], the attempts to apply this alternative treatment in SCA are still few. Okabe et al. (2007) reported a patient with SCA6 was treated with a mixture of 18 medical herbs (modified Zhengan Xifeng Tang) and then the patient's ataxia was remarkably reduced [29]. However, the therapeutically effective compound(s) in this remedy remains unknown. In this study we identified the aqueous extract of *P. lactiflora* that reduced ATXN3-aggregates mainly via its active compound paeoniflorin (Figures 2 and 3). The reporter gene assay and Western blotting further indicated the aggregation-reduction effect of paeoniflorin was modulated by the up-regulation of HSF1 and its targets, HSPA8 and HSPA1A chaperone expressions (Figure 4). 

*P. lactiflora*, with immunomodulatory and anti-inflammatory effects [30], has been widely used as a component of traditional Chinese prescriptions to relieve pain and to treat rheumatoid arthritis, systemic lupus erythromatosus, dysmenorrhea, hepatitis, muscle spasm, and fever with a long history. Its main bioactive component, paeoniflorin, possesses wide pharmacological effects in the nervous system. It has been reported to decrease the death of rat cortical cells while exposed to H_2_O_2_-induced oxidative stress [31]. Subcutaneous injection of paeoniflorin has shown functional protection in the 6-OHDA lesion rodent model of PD [16], and reduce the MPTP-induced toxicity by activation of the adenosine A1 receptor to inhibit neuroinflammation [15]. In A*β*_(1–42)_-injected AD rat model, paeoniflorin attenuates the neurotoxicity and cognitive decline by regulating calcium homeostasis and ameliorating oxidative stress [17]. Our results demonstrate both extract of *P. lactiflora* and paeoniflorin up-regulating HSF1 and HSP70 expressions to inhibit aggregate formation in ATXN3/Q_75_ 293 cells (Figure 5). This aggregation-inhibitory effect can also be seen in neuronal differentiated SH-SY5Y cells expressing ATXN3/Q_75_ (Figure 7), providing a novel mechanism of *P. lactiflora* and paeoniflorin to slow down the neurodegenerative process by inducing the expression of heat shock proteins.

Peoniflorin was the first reported active component of herbal medicines to induce expression of heat shock proteins in HeLa, IMR-32, and normal rat kidney cells [32]. Upon stress, HSF1 is released from the chaperone complex, self-trimerizes, and then is transported into the nucleus as a transcription factor. It activates chaperones, which play an important role in preventing unwanted protein aggregation. Overexpression of HSF1 significantly improved the life span of R6/2 Huntington's disease mouse [33]. Up-regulation of chaperone expression by HSF1 and its activating compounds 17-(allylamino)-17-demethoxygeldanamycin (17-AAG) demonstrate a strong inhibitory effect on HD aggregate formation [34]. We also showed aggregation-inhibitory effect of HSF1 overexpression in 293 cells expressing ATXN3/Q_75_ (Figure 6). Overexpression of its downstream target gene HSPA1A suppresses polyQ-mediated neurodegeneration in *Drosophila* and mouse models [35, 36]. Reduced expression of HSPA8, another target genes of HSF1 [37], has been shown in the 293 cells overexpressing expanded TBP-Q_61_ [38] and SCA17 lymphoblastoid cells [39]. The therapeutic potential of *P. lactiflora* and paeoniflorin in the treatment of SCA is strongly supported by the up-regulated HSF1 and HSP70 expressions.

In conclusion, our *in vitro* study provides strong evidence that *P. lactiflora* and paeoniflorin could be novel therapeutics for SCA3 and other polyQ diseases. Future application of *P. lactiflora* and paeoniflorin to SCA animal models would solidify their effects on aggregation reduction and disease improvement. Since the pathogenesis of the polyQ diseases is not completely clear and effective treatment is not available, our cell models are extremely valuable for identifying potential therapeutic targets in polyQ diseases. A systemic high-throughput screening of herbal and chemical compounds using ATXN3/Q_75_ 293 cell model is undergoing.
